# Effects of olive leaf extract supplementation on systemic markers of tissue aging and remodeling in postmenopausal women: a randomized controlled trial with exploratory skin outcomes

**DOI:** 10.3389/fnut.2025.1670194

**Published:** 2025-11-18

**Authors:** Anissa Lasfar, Sanne L. M. van Stratum, Maria Imperatrice, Colin A. J. van Kalkeren, Jean L. J. M. Scheijen, Casper G. Schalkwijk, Danique La Torre, Freddy J. Troost

**Affiliations:** 1 Solabia BV, Maastricht, Netherlands; 2 Department of Nutrition and Movement Sciences, Institute of Nutrition and Translational Research in Metabolism (NUTRIM), Maastricht University, Maastricht, Netherlands; 3 Department of Human Biology, Institute of Nutritional and Translational Research in Metabolism (NUTRIM), Maastricht University, Maastricht, Netherlands; 4 Cardiovascular Research Institute Maastricht (CARIM) School for Cardiovascular Diseases, Maastricht University, Maastricht, Netherlands; 5 Department of Internal Medicine, Maastricht University Medical Center+, Maastricht, Netherlands; 6 Food Innovation and Health, Centre for Healthy Eating and Food Innovation, Maastricht University, Venlo, Netherlands

**Keywords:** olive leaf extract, oleuropein, women's health, skin, pore number, surface skewness

## Abstract

**Introduction:**

Menopause marks the end of a woman's reproductive cycle and is associated with a decline in estrogen levels. This hormonal shift accelerates systemic aging processes, affecting metabolic regulation, cardiovascular risk, and connective tissue integrity. Circulating biomarkers offer a non-invasive way to monitor these changes.

**Objectives:**

This randomized, double-blind, placebo-controlled study aimed to determine the effects of 12 weeks of olive leaf extract (OLE) supplementation on systemic markers of tissue aging and remodeling in postmenopausal women (45–70 years), and explored skin quality in a subgroup.

**Methods:**

Sixty-five healthy postmenopausal women received 250 mg OLE or placebo daily. Circulating levels of elastin, collagen, hydroxyproline, matrix metalloproteinase-2 (MMP-2), advanced glycation end-products, and fasting glucose were measured. In a subgroup (*n* = 26), skin quality was assessed via video dermoscopy to explore the peripheral effects of OLE supplementation.

**Results:**

Elastin levels significantly increased in the placebo group while they remained stable in the OLE group [−6.3 [−12.0; −0.05], *p* = 0.033], but not after correction for multiple testing (*p*_adj_ = 0.0825). Pentosidine significantly decreased in the OLE group compared to placebo [−0.75 [−1.40; −0.11], *p* = 0.022], but also not after correction (*p*_adj_ = 0.088). Collagen, hydroxyproline, MMP-2, and glucose remained unaffected. In the exploratory skin analyses, pore number significantly decreased in the OLE group between weeks 6 and 12 [−12.9 [5.64; 20.16], *p* = 0.0012], while the placebo group showed no significant change [+1.25, [−6.99; 4.49], *p* = 0.657]. At week 12, the OLE group had a significantly lower pore number compared to placebo [−7.86, [0.64; 15.07], *p* = 0.034]. Surface skewness significantly decreased in the OLE group between weeks 6 and 12 [−0.32, [0.06; 0.58], *p* = 0.0166], while the placebo group showed no significant change [+0.1, [−0.31; 0.10], *p* = 0.3149]. At week 12, the OLE group showed a lower tendency toward surface skewness compared to placebo [−0.26, [−0.04; 0.56], *p* = 0.0847].

**Conclusion:**

The exploratory skin analyses revealed a reduction in pore number and surface skewness, suggesting that OLE may exert localized effects on skin structure. Although no statistically significant effects on systemic markers associated with tissue aging and remodeling were observed, the trends suggest potential modulation of pathways involved in extracellular matrix preservation and protein glycation. These findings warrant further investigation into both systemic and dermal effects of OLE in the context of postmenopausal aging.

**Clinical trial registration:**

The study was registered online at ClinicalTrials.gov as NCT05744453 and was conducted at Maastricht University.

## 1 Introduction

Menopause is a natural biological transition that marks the end of the female reproductive cycle. Usually occurring between the ages of 45 and 55, the menopausal transition begins with gradual or sudden cessation of estradiol and progesterone production by the ovaries. This hormonal shift has widespread effects on various tissues and organ systems. Beyond its reproductive implications, menopause is increasingly recognized as a turning point in systemic health, contributing to changes in metabolic regulation (1, 2), cardiovascular risk (3), and connective tissue integrity (4). The reduction in estrogen levels negatively impacts connective tissue components such as bone turnover (5–7), intervertebral discs (8, 9), and skin collagen content (10, 11).

As tissues age, structural and biochemical changes occur that affect their integrity, elasticity, and function. These changes are closely linked to the composition and turnover of the extracellular matrix (ECM), which plays a central role in maintaining tissue architecture. Systemic biomarkers offer a non-invasive means, providing a snapshot of age-related alterations in tissue composition and remodeling. For example, increased activity of matrix metalloproteinases (MMPs) indicates enhanced ECM breakdown (12), while the accumulation of advanced glycation end-products (AGEs) reflects oxidative stress and protein damage (13). By measuring circulating molecules involved in ECM degradation, protein modification, and metabolic regulation, it is possible to obtain a physiological snapshot of age-related tissue decline. This approach is particularly relevant in postmenopausal women, where hormonal shifts accelerate these systemic changes.

Olive leaf extract (OLE), derived from *Olea europaea*, is rich in polyphenolic compounds such as oleuropein and hydroxytyrosol, which have demonstrated potent antioxidant and anti-inflammatory properties (14–19). These bioactive compounds are known to counteract key mechanisms of tissue aging, including oxidative stress (20, 21) and protein glycation (22). OLE has shown promise in improving blood lipid profiles, indicating its potential for broader systemic effects. Clinical research on OLE supplementation, using products with a total polyphenol content between 167 and 400 mg (standardized for 100–200 mg oleuropein), has shown promising systemic effects, particularly on cardiovascular health. Across multiple studies, improvements have been reported in blood pressure, total cholesterol, low-density lipoprotein cholesterol, and triglyceride levels (23–25). Given its multifaceted biological activity, OLE may serve as a supportive intervention to mitigate age-related tissue decline, particularly in postmenopausal women experiencing accelerated aging processes.

A recent publication from the same randomized controlled trial showed that 12 weeks of OLE supplementation significantly improved postmenopausal symptoms as assessed by the validated Menopause-Specific Quality of Life Questionnaire. In addition, OLE supplementation had beneficial effects on bone mineral density in the right arm, and a decrease in serum triglyceride concentrations and the triglyceride/high-density lipoprotein cholesterol ratio as compared to the placebo group (26). These findings suggest that OLE may positively influence systemic aging processes. To further investigate these effects, we conducted additional analyses within the same trial, focusing on systemic markers associated with tissue aging and remodeling, including collagen, elastin, hydroxyproline, MMP-2, and AGEs. By focusing on circulating biomarkers, we sought to gain mechanistic insight into the physiological effects of OLE beyond symptom relief.

While systemic biomarkers offer insight into age-related physiological changes, they may not fully capture localized tissue responses. The skin, as a peripheral and highly dynamic organ, is particularly sensitive to oxidative stress and extracellular matrix remodeling, processes highly relevant during postmenopausal aging. Moreover, the skin is accessible for non-invasive assessment and has been proposed as a visible proxy for underlying tissue health. To explore whether OLE might exert localized effects independent of systemic changes, skin texture, tone, and structural features were analyzed in a subgroup of participants. This analysis was exploratory in nature and intended to complement the systemic findings, thereby generating hypotheses for future research. Together, these approaches address the current knowledge gap by linking OLE supplementation to both systemic biomarkers and localized skin outcomes, providing a more comprehensive understanding of its potential role in mitigating age-related tissue decline in postmenopausal women.

## 2 Material and methods

### 2.1 Study population

Healthy postmenopausal women (defined as amenorrhea over 12 months) aged 47–70 years were recruited through local advertisements and social media. Additionally, participants from our previous intervention studies were contacted if they had provided written consent for future contact. Interested participants were invited for a screening visit to evaluate their eligibility if they met the following criteria: body mass index (BMI) <35 kg/m^2^ and no use of hormone replacement therapy—either estrogenic or progestogenic- in the past 3 months. Additional inclusion criteria included: willingness to maintain their routine use of facial cream/treatment during the study period and to avoid overexposure to sunlight or tanning beds (solariums) on the tested area within 30 days prior to baseline and during the study (exposure after sunscreen application was allowed). Exclusion criteria included: use of antibiotics and/or supplements within 3 months before the start of the study (except for calcium and vitamin D with a constant daily dosage); allergy to the test or placebo product, olive leaves or olive oil; smoking; abuse of alcohol (>20 alcoholic units/week) or drugs; Botulinum toxin A (Botox) or filler (e.g., collagen, hyaluronic acid) injection treatments near the test areas within 2 years prior to baseline or planned during the study; history of breast cancer; history of gastric bypass surgery.

The study was approved by the Medical Ethics Committee of University Hospital Maastricht and Maastricht University (METC azM/UM) and conducted at Maastricht University in accordance with the 1964 Declaration of Helsinki and its later amendments. The study was registered at ClinicalTrials.gov as NCT05744453. Written informed consent was obtained from all participants. This trial was performed between January 2023 and May 2024. Data collection and management were overseen by an independent researcher to ensure integrity and compliance with the study protocol. All analyses were performed in a blinded manner. Investigators responsible for statistical analysis were unaware of group allocation until after the database was locked. This approach helped minimize bias and ensure objective interpretation of the results.

### 2.2 Study design

This 12-week randomized, double-blind, placebo-controlled, parallel trial was conducted with participants assigned to receive either OLE or placebo. The study visits were performed in temperature-controlled rooms at the Metabolic Research Unit Maastricht (MRUM). Randomization was performed by an independent researcher using the Castor Electronic Data Capture platform (Amsterdam, The Netherlands), with block randomization through the Castor integrated randomization tool. To ensure blinding, only this independent researcher had access to the randomization module and was able to view group allocations. Additionally, study procedures were overseen by an external monitor, who ensured compliance with Good Clinical Practice and verified that blinding and allocation procedures were correctly implemented. Participants visited the MRUM at baseline, and after 6 and 12 weeks. All study visits were performed in the morning after an overnight fast. Subjects were asked to maintain their dietary habits during the entire study period and to avoid strenuous physical activity and alcohol on the day prior to each test day. Moreover, participants were asked not to wear makeup or apply creams to their faces or forearms during each visit. On each test day, blood samples were collected from an antecubital vein in the forearm. In a subgroup of the study population (*n* = 26), skin quality was assessed with the C-Cube dermoscope (Pixience, Toulouse, France) as an exploratory outcome.

### 2.3 Intervention

The OLE (Bonolive^®^) was supplied by Solabia BV (Maastricht, The Netherlands). Bonolive^®^ consists of a mixture of polyphenols derived from olive leaves and is standardized to contain >40% oleuropein content. The polyphenol profile of the OLE is shown in Table 1. The remainder of the OLE consists of other major and minor components of olive leaves, such as carbohydrates, proteins, and minerals. The nutritional value of the OLE is shown in Table 2. The OLE and placebo capsules were indistinguishable in appearance, taste, and smell to maintain blinding. Participants consumed 250 mg of OLE (providing ≥100 mg oleuropein) or 250 mg of placebo (cellulose) daily with breakfast and a glass of water. Participants were asked to record the daily capsule intake, along with any deviations, in a supplementation logbook. The remaining capsules were collected at the end of the study period to assess compliance, which was considered valid if >80% of the study product had been consumed.

**Table 1:** Polyphenol profile of the OLE.

**Polyphenol**	**Relative percentage**
Hydroxytyrosol glucoside	0.17
Oleoside	3.25
Hydroxytyrosol	1.72
Oleoside-11-methyl ester (isomers 1 & 2)	3.59
Demethyloleuropein	0.97
Verbascoside	2.14
Rutin	0.60
Apigenin-7-glucoside	0.40
p-HPEA-EA (tyrosol derivative)	0.32
Oleuropein	83.88
Luteolin-7-glucoside	2.23
3,4-DHPEA-EA (hydroxytyrosol derivative)	0.54
Luteolin	0.19
Total	100.00

Quantitative results of polyphenol analysis may vary depending on the laboratory, techniques and methods, and variability in reference standards. Results should be considered quantitative.

**Table 2:** Nutritional value of the OLE.

**Description**	**Nutritional value per 100 grams of product**
Energy (kcal)	381.3
Total fat content (g)	0.3
Saturated fatty acids	0.17
Total carbohydrate content (g)	94.4
Assimilable carbohydrates (g)	93.1
Total sugars (as glucose) (g)	4.2
Total fibers (g)	1.3
Total protein (g)	0.9
Sodium (mg)	62.7

### 2.4 Biochemical analysis

Blood samples were taken after an overnight fast on each test day and collected in serum or plasma separator tubes (BD Vacutainer, NJ, USA). After blood collection, serum tubes were stored at room temperature for at least 30 min, and centrifuged within 1 h (20 °C, 1,300 × g, 10 min). Plasma tubes were stored on ice immediately after collection and centrifuged within 30 min (4 °C, 1,300 × g, 10 min). After centrifugation, serum and plasma aliquots were snap-frozen and stored at −80 °C until analysis at the end of the trial. Enzyme-linked immunosorbent assays (ELISAs) were used to measure serum levels of human elastin (Antibodies online GmbH, Aachen, Germany), pro-collagen type I alpha 1 (pro-C1α1) (Boster Biological Technology, CA, USA), plasma levels of hydroxyproline (Antibodies online GmbH, Aachen, Germany), and serum MMP-2 (Elabscience, Texas, USA) according to the manufacturer's protocol. Furthermore, plasma fasting glucose concentrations were measured on a Cobas Pentra C400 (Horiba ABX) using a commercially available kit: ABX Pentra Glucose HK CP (Horiba ABX Diagnostics, Montpellier, France), following the manufacturer's protocol. For all ELISA and fasting glucose measurements, assay validation was performed by assessing intra-assay variability. Only samples with a coefficient of variation below 10% were included in the final analysis. Samples exceeding this threshold were reanalyzed for confirmation of reliability and reproducibility.

Additionally, the concentration of the AGEs [N^ε^-(carboxymethyl)lysine (CML), N^ε^-(carboxyethyl)lysine (CEL) and N^δ^-(5-hydro-5-methyl-4-imidazolone-2-yl)-ornithine (MG-H1)] in plasma samples was measured with ultra-performance liquid chromatography tandem mass spectrometry (UPLC-MS/MS), as described in detail previously (27), at baseline and after 12 weeks. Furthermore, plasma pentosidine levels were measured with reversed-phase-high-performance liquid chromatography (HPLC) and fluorescence detection as described in detail previously (28), at baseline and after 12 weeks. Assay validation for AGE measurements was performed as described previously (27, 28). Lower limits of quantification (LLOQs) for protein-bound CML, CEL, and MG-H1 were 4, 4, and 7 fmol on column, corresponding to concentrations of 200, 200, and 340 nmol/L, respectively. For free CML, CEL, and MG-H1, the LLOQs were 4, 3, and 6 fmol on column, corresponding to concentrations of 8.5, 4.9, and 11.8 nmol/L, respectively. The limit of detection (LOD) for pentosidine was 2.2 nmol/L, which is equivalent to 0.02 pmol/mg protein. Samples with concentrations below the LLOQ or LOD were excluded or reanalyzed to ensure data reliability.

### 2.5 Skin quality assessment: subgroup analysis

Skin quality was assessed using the C-Cube video-dermoscope (Pixience, Toulouse, France) in a subgroup of the study population. All measurements were performed with subjects in a supine position after a 15-min skin acclimation period. The skin was ensured to be dry and clean, without makeup or cream. C-Cube video-dermoscope was deployed to take high-resolution 2D and 3D pictures on the face. Phototype was assessed via a photograph of the inner upper arm. Pictures were analyzed with the C-Cube Quickscale and C-Cube Clinical Trial Software (Pixience, Toulouse, France). Outcomes measured included skin pigmentation, pore number, surface skewness, wrinkle depth, and hair density. Moreover, facial hydration was assessed with the Hydration probe (Pixience, Toulouse, France).

### 2.6 Statistical analysis

The present manuscript is a secondary publication based on the same randomized controlled trial previously described, focusing on postmenopausal symptoms (26). The power calculation for the original trial was based on changes in appendicular fat-free mass. Based on an expected effect size of 0.91, a power of 0.90, and a two-sided alpha of 0.05, a minimum of 54 participants was required. Accounting for a 15% drop-out rate, at least 64 subjects were included. For the primary and secondary outcomes, correction for multiple testing was performed by the false-discovery rate (FDR) according to Benjamini–Hochberg, applied across multiple time points and parameters. An exploratory skin quality assessment was performed in a subgroup of 26 participants using the C-Cube. In a previous study that used the C-Cube dermoscope to evaluate multiple skin parameters, a significant effect on skin characteristics was observed after 8 weeks of intervention in a sample of 22 Caucasian women (45–70 years) (29). Given the exploratory nature of the sub-group measurements, no adjustment to the sample size or multiple testing correction was deemed necessary. All statistical analyses were performed using IBM SPSS Statistics (26.0, IBM Corporation, Armonk, NY, USA).

For the systemic markers and the skin quality outcomes, statistical analyses were performed using intention-to-treat linear mixed models including treatment and time as fixed factors, baseline values as covariates, and participant as random factors with random intercepts. Non-significant time × treatment interactions were removed from the models. In cases where a significant time × treatment interaction was observed, *post-hoc* analyses using estimated marginal means and pairwise comparisons were conducted to further explore these effects. Data are reported as unadjusted means ± SDs, unless otherwise specified. A *p*-value ≤ 0.05 was considered statistically significant. *P*-values ≤ 0.09 were considered a trend.

## 3 Results

### 3.1 Participants

**Figure 1:**
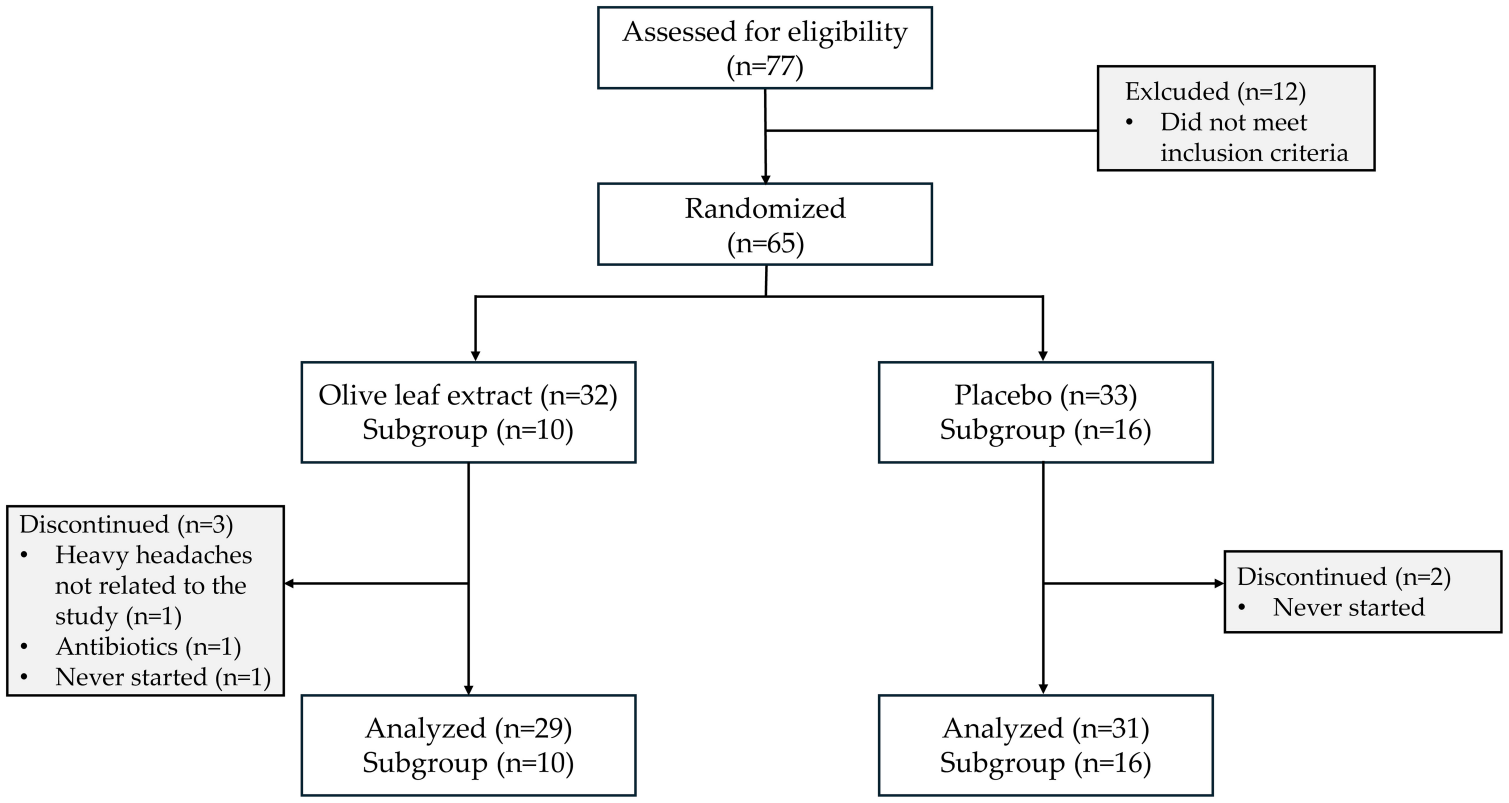
CONSORT diagram. Flow chart showing participant screening, exclusion, randomization, subgroups, follow-up, and analysis.

In total, 77 participants were screened for eligibility, of which 65 were included in the current study. Three participants allocated to the OLE group discontinued the study, one after the baseline measurements due to health reasons not related to the study (severe headaches), another after 6 weeks because of antibiotic intake, and one did not start the intervention after randomization. In the placebo group, two participants who were allocated never started the study after randomization. In the subgroup for the evaluation of skin quality with the C-Cube video-dermoscope, 26 participants were included; 10 were randomized into the OLE group and 16 to the placebo group (Figure 1).

### 3.2 Baseline characteristics

Baseline characteristics of the study population are presented in Table 3. The study product was well-tolerated, and no serious adverse events or protocol deviations were reported.

**Table 3:** Baseline characteristics of the olive leaf extract and placebo group^a^.

**Characteristic**	**Total population (*n* = 60)**	**OLE (*n* = 29)**	**Placebo (*n* = 31)**	**Subgroup OLE (*n* = 10)**	**Subgroup placebo (*n* = 16)**
Age (years)	59.2 ± 5.8	58.4 ± 6.2	59.8 ± 5.4	56.9 ± 5.8	59.8 ± 6.8
Height (cm)	165.0 ± 6.7	165.5 ± 7.2	165.2 ± 6.3	165.2 ± 4.5	163.3 ± 5.6
Weight (kg)	69.7 ± 9.6	68.5 ± 8.6	70.8 ± 10.5	72.2 ± 7.9	67.8 ± 9.2
BMI kg/m^2^	25.4 ± 2.8	24.9 ± 2.1	25.9 ± 3.4	26.4 ± 1.9	25.5 ± 3.7
Phototype	–	–	–	1.8 ± 1.5	1.8 ± 0.6

^a^Values are presented as mean ± SD. BMI, Body Mass Index; OLE, olive leaf extract.

### 3.3 Biochemical analysis

Results of biochemical analyses performed on blood samples are reported in Table 4. Elastin degradation significantly increased in the placebo group compared to the OLE group after 6 and 12 weeks [−6.3 [−12.0; −0.5] *p* = 0.033], but not after correction for multiple testing (*p*_adj_ = 0.0825). Collagen, hydroxyproline, MMP-2, and fasting glucose remained unaffected (Table 4).

**Table 4:** Biochemical analysis following the OLE and placebo intervention period in postmenopausal women^a^.

**Outcome**	**OLE intervention (*****n*** = **29)**			**Placebo (*****n*** = **31)**			**Treatment effect (OLE-placebo)**	**Adjusted *P*-value (FDR)**
	**T0**	**T6**	**T12**	**T0**	**T6**	**T12**		
Serum elastin (ng/mL)	69.0 ± 18.9	67.2 ± 16.3	68.5 ± 20.4	73.3 ± 19.6	75.7 ± 18.7	80.5 ± 25.4	−6.3 [−12.0; −0.5]; *p* = 0.033^*^	*p*_adj_ = 0.0825
Serum pro-C1α1 (ng/mL)	368.9 ± 163.5	379.7 ± 184.4	379.4 ± 165.8	381.1 ± 159.8	402.5 ± 158.5	422.3 ± 154.3	−27.8 [−73.0; 18]; *p* = 0.229	*p*_adj_ = 0.286
Plasma hydroxyproline (μg/mL)	1.2 ± 0.7	1.3 ± 0.8	1.2 ± 0.8	1.1 ± 0.8	1.0 ± 0.7	1.1 ± 0.8	−0.067 [−0.210; 0.072]; *p* = 0.338	*p*_adj_ = 0.338
Serum MMP-2 (ng/mL)	357.0 ± 120.4	345.6 ± 100.9	345.5 ± 102.6	392.5 ± 122.5	384.1 ± 126.6	406.4 ± 132.2	0.334 [−22.3; 23.0]; *p* = 0.997	*p*_adj_ = 0.997
Fasting glucose (mmol/L)	6.0 ± 0.63	6.0 ± 0.90	6.2 ± 0.80	5.9 ± 0.84	6.0 ± 0.78	6.1 ± 0.76	−0.015 [−0.28; 0.26]; *p* = 0.913	*p*_adj_ = 0.997

^a^Values are presented as means ± SDs. Differences between OLE and placebo were calculated with a linear mixed model analysis with random intercept. Differences between OLE and placebo were calculated with a linear mixed model analysis with random intercept. Time × treatment was used as a fixed factor, and participant as a random factor. *P*-values for the treatment effect between OLE and placebo intervention (mean difference [95% CI]) were reported. ^*^ Indicates *p* ≤ 0.05. FDR, False discovery rate; MMP-2, matrix metalloproteinase 2; OLE, olive leaf extract; pro-c1α1, Collagen type I alpha 1.

### 3.4 Advanced glycation end products

Pentosidine significantly decreased in the intervention group compared to placebo after 12 weeks [−0.751 [−1.4; −0.11], *p* = 0.022], but not after correction for multiple testing (*p*_adj_ = 0.088). The other measured AGEs, CML, CEL and MG-H1 remained unaffected (Table 5).

**Table 5:** protein bound advanced glycation end products in plasma in nmol/L following the OLE and placebo intervention period in postmenopausal women.

**Outcome**	**OLE intervention (*****n*** = **29)**		**Placebo (*****n*** = **31)**		**Treatment effect (OLE—placebo)**	**Adjusted *P*-value (FDR)**
	**T0**	**T12**	**T0**	**T12**		
CML (nmol/L)	3315.9 ± 645.2	3229.1 ± 572.9	3366.8 ± 607.2	3345.8 ± 569.7	−82.5 [−290.3; 125.3]; *p* = 0.429	*p*_adj_ = 0.572
CEL (nmol/L)	1133.4 ± 645.2	1146.0 ± 306.6	1215.8 ± 450.2	1123.9 ± 319.7	59.3 [−78.7; 19.4]; *p* = 0.393	*p*_adj_ = 0.786
MG-H1 (nmol/L)	675.5 ± 110.0	692.5 ± 244.3	700.4 ± 110.9	688.4 ± 99.5	25.3 [−59.5; 110.1]; *p* = 0.552	*p*_adj_ = 0.552
Pentosidine (nmol/L)	8.9 ± 3.2	8.1 ± 2.5	8.4 ± 2.1	8.5 ± 2.5	−0.751 [−1.4; −0.11]; *p* = 0.022^*^	*p*_adj_ = 0.088

CML, N^ε^-(carboxymethyl)lysine; CEL, N^ε^-(carboxyethyl)lysine; FDR, False discovery rate; MG-H1, N^δ^-(5-hydro-5-methyl-4-imidazolone-2-yl)-ornithine; OLE, olive leaf extract. ^*^ Indicates *p* < 0.05 statistical significance.

### 3.5 Skin quality (subgroup analysis)

**Figure 2:**
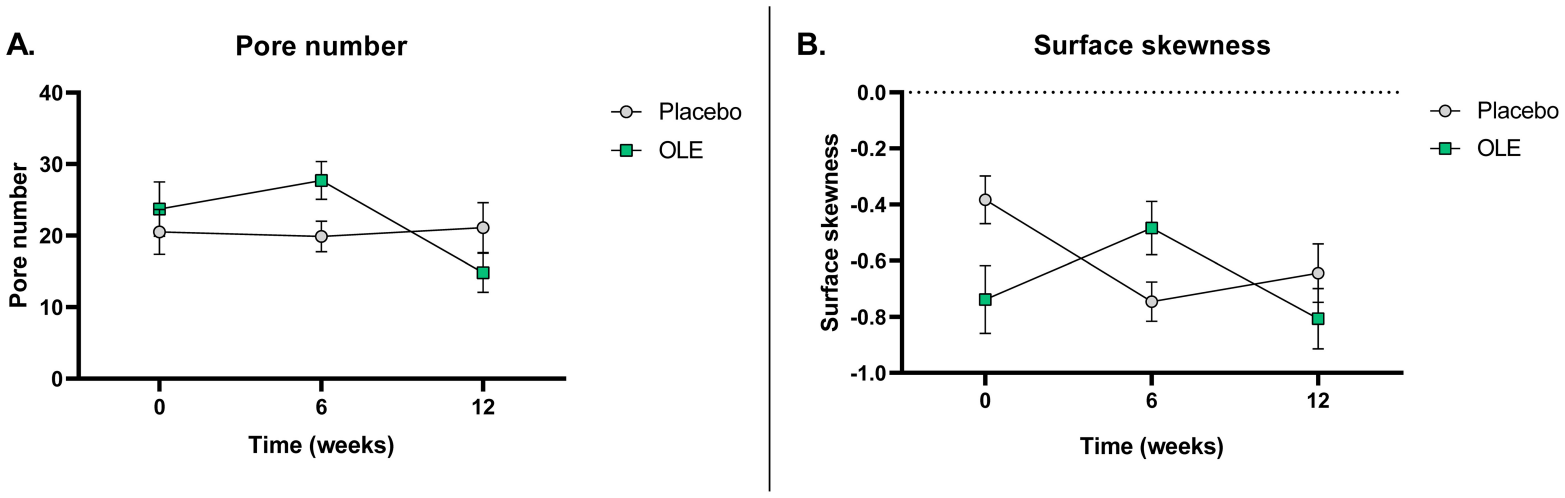
Pore number **(A)** and surface skewness **(B)** at baseline, after 6 and 12 weeks of placebo vs. OLE supplementation in postmenopausal women assessed with a C-Cube video dermoscope (*n* = 26). Surface skewness represents the degree of symmetry of the surface heights about the mean plane. Analyses were performed with an intention-to-treat linear mixed model using time and treatment as a fixed factor, baseline values as covariates, and participant as random factors with random intercepts. *Post-hoc* estimated marginal means were analyzed and pairwise comparisons were made. OLE, Olive leaf extract. Raw data are shown as mean ± SEM.

The results of the skin quality assessments using C-Cube and analyzed with the Quickscale software are reported in Table 6. A significant time × treatment interaction for pore number (*p* = 0.004) was observed. *Post-hoc* analysis of the estimated marginal means and pairwise comparisons revealed a significant reduction in pore number in the OLE group after 6 weeks of intervention compared to 12 weeks [−12.9, [5.64; 20.16], *p* = 0.0012], while the placebo group showed no significant change [+1.25, [−6.99; 4.49], *p* = 0.657]. Additionally, after 12 weeks, the OLE group had a significantly lower pore number compared to the placebo group [−7.86, [0.64; 15.07], *p* = 0.034] (Figure 2). All other outcomes remained unaffected.

**Table 6:** Quickscale software skin measurements performed on the cheek following the OLE and placebo intervention period in a sub group of postmenopausal women^a^.

**Outcome**	**OLE intervention (*****n*** = **10)**			**Placebo (*****n*** = **16)**			**Time × treatment interaction**	**Treatment effect (OLE—Placebo)**
	**T0**	**T6**	**T12**	**T0**	**T6**	**T12**		
Redness (%)	0.3 ± 0.0	0.2 ± 0.0	0.2 ± 0.0	0.2 ± 0.0	0.2 ± 0.0	0.2 ± 0.0	*p* = 0.208	−0.015 [−0.04; 0.01]; *p* = 0.318
Evenness (%)	0.7 ± 0.1	0.8 ± 0.0	0.7 ± 0.1	0.8 ± 0.0	0.8 ± 0.0	0.7 ± 0.0	*p* = 0.877	−0.034 [−0.07; 0.00]; *p* = 0.101
Pigmentation (%)	0.1 ± 0.0	0.1 ± 0.0	0.2 ± 0.0	0.2 ± 0.0	0.2 ± 0.0	0.2 ± 0.0	*p* = 0.294	−0.010 [−0.04; 0.02]; *p* = 0.558
Forehead wrinkles depth (μm)	169.5 ± 54.6	210.3 ± 99.3	180.9 ± 63.9	159 ± 71.9	194.7 ± 99.5	171.9 ± 134.9	*p* = 0.857	+ 3.4 [−54; 61]; *p* = 0.904
Forehead wrinkles volume (mm3)	1.0 ± 0.6	1.9 ± 1.5	1.6 ± 0.9	1.8 ± 1.9	1.9 ± 1.7	1.9 ± 1.8	*p* = 0.668	+0.29 [−0.69; 1.2]; *p* = 0.544
Crowfeet wrinkles depth (μm)	85.9 ± 10.4	122.7 ± 64.6	200.1 ± 108.1	158.8 ± 82.6	133.5 ± 82.6	161.6 ± 112.3	*p* = 0.346	+46 [−2.5; 94]; *p* = 0.062
Crowfeet wrinkles volume (mm3)	0.5 ± 0.5	1.0 ± 1.2	2.1 ± 1.5	0.8 ± 0.7	1.3 ± 2.3	1.3 ± 1.6	*p* = 0.219	+0.67 [−0.45; 1.8]; *p* = 0.232
Texture average roughness (Sa) (μm)	19.4 ± 5.8	23.1 ± 6.9	21.4 ± 4.6	24.3 ± 6.8	21.3 ± 6.9	21.8 ± 6.5	*p* = 0.403	+3.2 [−0.41; 6.9]; *p* = 0.079
Pores depth (μm)	49.8 ± 15.5	39.6 ± 9.1	49.7 ± 20.5	43.4 ± 16.2	42.6 ± 14.1	46.6 ± 25.0	*p* = 0.556	−2.9 [−12; 6.5] *p* = 0.522
Pores diameter (μm)	15.0 ± 1.1	15.5 ± 0.8	15.5 ± 1.9	15.7 ± 1.3	16.3 ± 1.3	15.3 ± 2.2	*p* = 0.345	−0.48 [−1.2; 0.30]; *p* = 0.217
Pores number	23.7 ± 12.0	27.7 ± 8.4	14.8 ± 8.6	20.5 ± 12.5	19.8 ± 8.5	21.1 ± 13.9	*p* = 0.004^*^	N/A
Hair density (hair/cm^2^)	75.7 ± 13.8	86.1 ± 24.0	95.5 ± 31.0	83.2 ± 26.4	82.5 ± 15.6	78.3 ± 15.1	*p* = 0.148	+7.8 [−6.9; 22.6]; *p* = 0.286
Mean hair diameter (μm)	63.4 ± 19.6	68.6 ± 14.9	74.7 ± 10.0	66.6 ± 22.1	72.1 ± 18.9	69.0 ± 26.4	*p* = 0.406	−0.21 [−12; 11]; *p* = 0.970
Min hair diameter (μm)	81.0 ± 22.7	92.8 ± 36.4	58.0 ± 18.9	69.6 ± 22.0	91.6 ± 39.3	74.6 ± 17.7	*p* = 0.275	−8.2 [−50; 34]; *p* = 0.400
Max hair diameter (μm)	98.1 ± 22.9	97.5 ± 29.0	98.2 ± 16.5	97.9 ± 27.7	106.7 ± 23.4	108.7 ± 20.9	*p* = 0.931	−9.9 [−21; 1.3]; *p* = 0.081

^a^Values are presented as means ± SDs. Differences between OLE and placebo were calculated with a linear mixed model analysis with random intercept. Time × treatment was used as fixed factors, and participant as random factor. If the interaction term was not significant, it was removed from the model. Mean difference [95% CI] and *p*-values were reported. N/A, not applicable; OLE, olive leaf extract; Sa, texture average roughness. ^*^ Indicates *p* < 0.05 statistical significance.

The results of the skin quality assessment using the C-Cube and analyzed with the Clinical Trial software are reported in Table 7. A significant time × treatment interaction for surface skewness (*p* = 0.014) was observed. *Post-hoc* analysis of the estimated marginal means and pairwise comparisons revealed a significant decrease in surface skewness in the OLE group after 6 weeks of intervention compared to 12 weeks [−0.32, [0.06; 0.58], *p* = 0.0166], while the placebo group showed no significant change [+0.1, [−0.31; 0.10], *p* = 0.3149]. Additionally, after 12 weeks, the OLE group tended to have a lower surface skewness than the placebo group [−0.26, [−0.04; 0.56], *p* = 0.0847] (Figure 2). No significant treatment effects were observed for the other outcomes.

**Table 7:** C-Cube Clinical Trial software skin measurements performed on the cheek following the OLE and placebo intervention period in a sub group of postmenopausal women^a^.

**Outcome**	**OLE intervention (*****n*** = **10)**			**Placebo (*****n*** = **16)**			**Time × treatment interaction**	**Treatment effect (OLE-placebo)**
	**T0**	**T6**	**T12**	**T0**	**T6**	**T12**		
**3D Cheek**								
Elevation average (μm)	−6.2 ± 2.5	−5.9 ± 2.5	−6.9 ± 2.1	−4.3 ± 2.5	−6.9 ± 2.3	−5.7 ± 3.3	*p* = 0.131	0.1 [−1.6; 2.0]; *p* = 0.854
Elevation max (μm)	132.4 ± 36.4	140.5 ± 34.2	129.2 ± 20.3	152.3 ± 38.0	137.8 ± 39.9	135.7 ± 35.9	*p* = 0.473	4.0 [−18.3; 26.4]; *p* = 0.710
Elevation min (μm)	−258.8 ± 60.9	−223.9 ± 20.2	−237.4 ± 34.6	−233.8 ± 42.3	−257.2 ± 52.4	−246.3 ± 46.6	*p* = 0.316	20.5 [6.6; 47.8]; *p* = 0.132
Developed interfacial area ratio (Sdr)	0.9 ± 0.2	0.8 ± 0.2	0.8 ± 0.1	0.9 ± 0.2	0.8 ± 0.2	0.8 ± 0.2	*p* = 0.140	0.0 [−0.1; 0.1]; *p* = 0.959
Average roughness (Sa) (μm)	31.8 ± 8.7	33.8 ± 6.3	30.5 ± 4.3	34.1 ± 7.8	32.3 ± 5.8	34.2 ± 9.2	*p* = 0.085	0.5 [−2.9; 4.0]; *p* = 0.753
Root mean square roughness (Sq) (μm)	42.3 ± 10.7	44.0 ± 7.1	40.5 ± 5.3	44.4 ± 9.0	43.0 ± 7.0	44.6 ± 10.6	*p* = 0.133	0.0 [−4.2; 4.2]; *p* = 0.999
Surface skewness (Ssk)	−0.7 ± 0.3	−0.4 ± 0.3	−0.8 ± 0.3	−0.3 ± 0.3	−0.7 ± 0.2	−0.6 ± 0.4	*p* = 0.014^*^	N/A
Kurtosis of the texture (Sku)	5.3 ± 1.4	4.3 ± 0.9	5.3 ± 2.0	4.3 ± 1.3	5.2 ± 1.3	4.7 ± 1.7	*p* = 0.098	−0.5 [−1.4; 0.4]; *p* = 0.261
Max height of the surface (Sz) (μm)	391.3 ± 83.5	364.5 ± 42.3	366.7 ± 46.8	386 ± 64.5	395 ± 76.6	382.0 ± 72.2	*p* = 0.625	−23.4 [−64; 17]; *p* = 0.244
Max peak height (Sp) (μm)	138.7 ± 37.8	146.5 ± 33.5	136.1 ± 21.0	156.7 ± 38.6	144.7 ± 39.6	141.4 ± 35.6	*p* = 0.592	3.8 [−18; 25]; *p* = 0.720
Max valley depth (Sv) (μm)	252.5 ± 59.8	217.9 ± 20.4	230.5 ± 35.0	229.5 ± 42.6	250.3 ± 52.4	240.6 ± 45.9	*p* = 0.883	−20 [−47; 6.5]; *p* = 0.129

^a^Values are presented as means ± SDs. Differences between OLE and placebo were calculated with a linear mixed model analysis with random intercept. Time × treatment were used as fixed factors, and participant as random factor. If the interaction term was not significant, it was removed from the model. Mean difference [95% CI] and *P*-values were reported. N/A, not applicable; OLE, olive leaf extract; Sa, average roughness; Sdr, developed interfacial area ratio; Sku, kurtosis of the texture; Sp, max peak height; Sq, root mean square roughness; Ssk, surface skewness; Sy, max valley depth; Sz, max height of the surface. ^*^ Indicates *p* < 0.05 statistical significance.

## 4 Discussion

In this randomized, double-blind, placebo-controlled trial, we investigated the effect of 12 weeks of OLE supplementation on systemic markers of tissue aging and remodeling in postmenopausal women. While no significant changes were observed in collagen, hydroxyproline, MMP-2, or glucose, elastin levels showed stabilization in the OLE group, whereas they declined in the placebo group. Similarly, a reduction in plasma pentosidine was observed in the OLE group compared to placebo. However, both effects lost statistical significance after correction for multiple testing. Additionally, exploratory skin analyses revealed improvements in pore number and surface skewness after OLE supplementation, suggesting potential local effects on skin morphology. Other skin quality outcomes (wrinkles, redness, etc.), were not affected.

Although the stabilization of elastin did not remain statistically significant after correction for multiple testing, this trend may point to a stabilization of elastin breakdown. Elastin is a key structural protein present in many tissues, including connective tissue, muscles, blood vessels, lungs, and skin. Its degradation is a natural part of aging and occurs slowly, with a half-life of approximately 70 years (30). Given this long turnover rate, short-term interventions may not lead to significant decreases in circulating elastin levels, even if degradation processes are affected. Since elastin levels in the current study were measured in blood, it remains uncertain which specific tissue(s) contributed to the observed changes. Nevertheless, increased elastin degradation in any tissue may affect systemic function by compromising the elasticity and function of vital organs, such as the skin, heart, and lungs, potentially contributing to cardiovascular or pulmonary diseases (31, 32). Hence, OLE may contribute to the preservation of tissue integrity by inhibiting or slowing down elastin degradation, which in turn could help maintain the structural and functional properties of tissues and organs such as connective tissue, muscles, and the skin. While the observed trend in elastin stabilization in the OLE group is not statistically significant, it may still reflect subtle biological effects that warrant further investigation in longer-term and tissue-specific studies. To our knowledge, no clinical evidence on the effect of olive polyphenols on elastin levels is currently available. However, *ex vivo* stimulation of isolated aortic segments from *Fbn*^1*C*1039*G*+/−^ mice, a model of elastin fragmentation, with hydroxytyrosol did not affect elastin content (33). In contrast, in a 3D full-thickness pigmented skin model, exposed to UV radiation, a mixture of olive (0.001%), pomegranate (0.01%), and osmanthus (0.01%) extracts repaired the elastin decrease induced by UV exposure (34). These findings suggest that while hydroxytyrosol alone may not affect elastin in vascular tissue, combinations of polyphenols may exert protective effects in skin models. Thus, the potential of OLE to preserve tissue integrity by slowing elastin degradation warrants further investigation, particularly in clinical tissue-specific contexts such as the skin.

Elastin degradation is closely linked to the activity of MMPs, particularly MMP-2 and MMP-9, in the skin. These enzymes are upregulated during aging and by external factors such as UV exposure, leading to diminished skin elasticity and increased wrinkle formation (35–37). Although MMP-2 was not affected in the present study, other studies have demonstrated that MMP activity can be inhibited by components present in OLE, such as oleuropein (38–40). Li et al. showed that oleuropein and its metabolite hydroxytyrosol inhibited elastase activity in cultured human skin fibroblasts (38). Additionally, Kimura and Sumiyoshi reported reduced expression of MMP-2, MMP-9, and MMP-13 in UVB-exposed mice treated with OLE (40). Furthermore, human keratinocytes and fibroblasts exposed to blue LED irradiation (photoaging) and pretreated with hydroxytyrosol were protected due to reduced MMP-1 and increased collagen type I expression compared to control (39). These findings suggest that OLE may modulate MMP activity and thereby inhibit elastin degradation, although this was not confirmed in the present study. The discrepancy between these findings and our results may be explained by several factors. First, many of the cited studies were conducted under stress or inflammatory conditions (UV exposure, oxidative stress), which strongly upregulate MMP expression and may enhance the observable effects of the components present in OLE. In contrast, our study population consisted of generally healthy postmenopausal women, and baseline MMP-2 levels may have been relatively low, limiting the potential for measurable reductions. Second, the concentration of active metabolites reaching systemic circulation or skin tissue may have been lower than in preclinical models, where higher doses or direct application are often used. These considerations highlight the need for future studies to investigate tissue-specific effects and to explore whether OLE's impact on MMPs is context-dependent.

Of the AGEs measured, a reduction in plasma pentosidine was observed in the OLE group compared to placebo, but this effect also lost significance after correction for multiple testing. AGEs are formed through non-enzymatic reactions between sugars and proteins, lipids, or nucleic acids, leading to stable and often irreversible molecular changes. Pentosidine levels are elevated in patients with metabolic syndrome compared to healthy individuals (41). A reduction in pentosidine levels has been shown to be associated with improved metabolic control and a lower risk of microvascular complications, hypertension, and hyperlipidemia (41–43). Thus, reduced pentosidine may reflect improved metabolic control. Although fasting glucose levels remained unaffected, triglyceride levels were significantly reduced in the OLE group compared to control, as reported in our previous article based on the same study population (26). In the skin, AGEs accumulate over time and are known to impair skin structure and function by promoting collagen cross-linking, reducing elasticity, and increasing oxidative stress and inflammation (44). Preclinical findings show that OLE can inhibit the formation of AGEs such as pentosidine in food models (45). However, given the lack of clinical evidence on glycation modulation by olive polyphenols, further mechanistic studies are needed to confirm whether OLE can influence glycation pathways *in vivo*.

Inflammation and oxidative stress also contribute to increased elastase activity. Postmenopausal estrogen decline is thought to contribute to increased inflammatory activity, a hallmark of inflammaging (46). Oleuropein is known for its anti-inflammatory properties. Asghariazar et al. demonstrated anti-inflammatory effects of oleuropein on human fibroblasts (47). In a study by Nobile et al., daily supplementation with a polyphenol-enriched dietary supplement containing oleuropein and hydroxytyrosol for 12 weeks in women exposed to air pollution showed reduced oxidative stress, reflected by a reduction in malondialdehyde in the skin stratum corneum and an increase in total antioxidant capacity (48). In the present study, subgroup analysis revealed improvements in pore number and surface skewness, suggesting structural skin improvements after OLE treatment. While we did not find a reduction in elastin, the observed trend in elastin stabilization suggests that OLE supplementation might improve skin structure by decreasing oxidative stress and thereby reducing elastase enzyme activity. However, the study was not powered to detect changes in skin quality; thus, further mechanistic studies are needed to substantiate these hypotheses.

The exploratory skin analyses revealed a significant reduction in pore number in the OLE group from week 6 to week 12, while the placebo group showed no significant change during this period. Additionally, after 12 weeks of intervention, participants receiving OLE had a significantly lower pore number compared to those receiving placebo. Pore number reflects skin surface microstructure and may be influenced by hormones, sebum production, hydration, and epidermal remodeling (49, 50). A reduction in pore number may indicate smoother skin morphology or improved barrier function.

Additionally, surface skewness decreased significantly in the OLE group between 6 and 12 weeks of the OLE intervention, while the placebo group showed no significant change. After 12 weeks, the OLE group had a lower tendency for surface skewness compared to the placebo group. Surface skewness is commonly used to assess skin texture, and describes the asymmetry of the skin surface in terms of height variations, comparing the distribution of peaks and valleys relative to the average plane. Positive values indicate a surface dominated by peaks, while negative values reflect more valleys or indentations.

Although a decrease in skewness is sometimes associated with acne scarring or pitting, in this context, we hypothesize that the observed reduction more likely reflects a smoother and more even skin texture, characterized by fewer prominent peaks and a more uniform surface (51). This interpretation is supported by the observed reduction in pore number at the same time points, which similarly suggests improved skin morphology. Interestingly, while fewer pores suggest fewer superficial valleys, the shift toward more negative skewness may also reflect a flattening of prominent surface peaks. Together, these changes could result in a more uniform skin topography, highlighting the complexity of interpreting skewness in relation to specific morphological features. Further analysis of skin microrelief is warranted to disentangle these effects and refine the interpretation of skewness metrics. While studies investigating the effect of oral OLE supplementation on skin health are scarce, topical application of OLE-enriched creams has been shown to support skin rejuvenation by affecting hydration, wrinkles, texture, and transepidermal water loss (52). Together, these findings indicate that OLE may exert local effects on skin morphology even in the absence of measurable systemic changes. This study provided valuable insights for future, more targeted investigations in postmenopausal women. However, due to the exploratory nature of the analysis and the associated increased risk of type I error, larger and sufficiently powered trials will be needed to confirm these results.

While the bioavailability and pharmacokinetics of the active compounds in OLE were not assessed in the present study, previous research using the same OLE demonstrated rapid absorption and systemic distribution of OLE-derived metabolites. García-Villalba et al. showed that after ingestion of 250 mg Bonolive^®^, metabolites such as hydroxytyrosol glucuronide, hydroxytyrosol sulfate, and oleuropein aglycone glucuronide appeared in the plasma of postmenopausal women within 30–75 min, with peak concentrations reached within the first hour. Importantly, postmenopausal women exhibited higher plasma concentrations of several OLE metabolites compared to premenopausal women, indicating that hormonal status may influence OLE bioavailability (53). These findings support the plausibility of systemic effects of OLE supplementation and provide a pharmacokinetic basis for interpreting the outcomes of the current study. While tissue-specific distribution (e.g., accumulation in skin) remains to be elucidated, the existing data confirm that the administered dose results in measurable systemic exposure.

A major strength of this study is its randomized, placebo-controlled design, which enhances the reliability of the findings. The research specifically targeted postmenopausal women, a population more susceptible to skin issues due to the decline in estrogen associated with menopause. While this narrowly defined cohort, comprising healthy, non-obese, non-smoking women, enhances internal validity, it also limits the generalizability of the findings to more diverse populations. This includes women from different ethnic backgrounds, those with comorbidities common in menopause, and individuals exposed to varying environmental factors globally, all affecting tissue aging and remodeling and skin health.

Although the findings of the present study suggest potential benefits of OLE, several limitations should be considered when interpreting the results. The systemic biomarkers assessed, including elastin, AGEs, collagen, hydroxyproline, and MMP-2, are not organ-specific and may originate from various tissues, limiting their utility in evaluating specific effects such as skin aging. Moreover, given the gradual nature of skin aging, a 12-week intervention may not be long enough to detect meaningful long-term effects. Future studies should include longer intervention periods and direct assessments of skin tissue, such as biopsies or advanced non-invasive imaging. Skin-specific measurements, such as measurements of lipid peroxides, moisturization, transepidermal water loss, skin radiance and color, elasticity and firmness, and sebum content as used in similar trials (48), would allow for more precise evaluation of dermal remodeling and provide clearer insight into the underlying mechanisms involved. In addition, future studies could consider stratification by menopausal stage (early vs. late postmenopause), as hormonal and metabolic differences across these phases may influence individual responses to OLE supplementation.

Additionally, lifestyle factors such as diet, UV exposure, and physical activity were self-reported and not quantitatively assessed, introducing variability that may have influenced the outcomes. These factors are known to affect skin health and aging, and their subjective reporting limits the ability to control for potential confounding effects. While the study employed a placebo-controlled design, it did not include a positive control such as a topical antioxidant or established skin intervention. Given that the primary aim of the main study was to investigate postmenopausal symptoms (26), the inclusion of a positive control specifically targeting skin outcomes was beyond the present sub-study's scope. Nevertheless, future studies focusing more directly on skin health could benefit from incorporating such comparators to enhance clinical relevance and interpretability of the findings.

## 5 Conclusion

This randomized, placebo-controlled trial did not demonstrate statistically significant effects of 12 weeks of OLE supplementation on systemic markers of tissue aging and remodeling in postmenopausal women. However, the observed trends in elastin preservation and pentosidine reduction suggest that OLE supplementation may influence pathways involved in extracellular matrix preservation and protein glycation. Additionally, exploratory skin analyses revealed improvements in pore number and surface skewness, suggesting localized effects on skin structure. These preliminary findings warrant further investigation into both systemic and dermal effects of OLE in the context of postmenopausal aging. Future studies should include direct skin assessments, larger sample sizes, and longer intervention periods to better characterize the therapeutic potential and underlying mechanisms of OLE.

## Data Availability

The raw data supporting the conclusions of this article will be made available by the authors, without undue reservation.
